# Application of ZnO NPs, SiO_2_ NPs and Date Pollen Extract as Partial Substitutes to Nitrogen, Phosphorus, and Potassium Fertilizers for Sweet Basil Production

**DOI:** 10.3390/plants13020172

**Published:** 2024-01-08

**Authors:** El-Sayed Mohamed El-Mahrouk, Ekramy Abdel Moatamed Atef, Mohamed Kadry Gabr, Mahmoud Ahmed Aly, Aleksandra Głowacka, Mohamed A. A. Ahmed

**Affiliations:** 1 Horticulture Department, Faculty of Agriculture, Kafr El-Sheikh University, Kafr El-Sheikh 33516, Egypt; elsayedelmahrouk@gmail.com; 2 Plant Production Department (Horticulture—Medicinal and Aromatic Plants), Faculty of Agriculture (Saba Basha), Alexandria University, Alexandria 21531, Egypt; ekramyatef@alexu.edu.eg; 3 Plant Production Department, Faculty of Agriculture (Saba Basha), Alexandria University, Alexandria 21531, Egypt; m_kadry@alexu.edu.eg (M.K.G.); dr_mahmoud_aly@hotmail.com (M.A.A.); 4 Department of Plant Cultivation Technology and Commodity Sciences, University of Life Sciences in Lublin, 13 Akademicka Street, 20-950 Lublin, Poland

**Keywords:** date pollen, fertilization, SiO_2_ NPs, sweet basil plant, ZnO NPs

## Abstract

The reduction in mineral fertilizer usage is crucial to the production of medicinal and aromatic products for safety and health purposes. Presently, nanotechnology and the utilization of natural extracts have been extensively studied due to their significant contribution. *Ocimum basilicum* is commonly employed for various medicinal and aromatic applications. Therefore, randomized complete block design field experiments containing 10 treatments were conducted during the 2021 and 2022 seasons to investigate the effect of nanoparticles (NPs) of ZnO (1.5 and 2.0 g/L) and SiO_2_ (100 and 150 mg/L) and date palm pollen extract (DPPE) at 10 and 20 g/L either alone or in combination with the ¾ or ½ NPK recommended dose (RD). The NPK RD was served as a control treatment on basil plant production in each season. The effectiveness of ZnO NPs, SiO_2_ NPs, and DPPE for the decrease in NPK utilization was evaluated. Meanwhile, the most effective treatment for vegetative traits (except for plant height), essential oil %, and yield was ½ NPK RD + 20 g/L DPPE + 2.0 g/L ZnO NPs. Such a treatment increased the branch number/plant, main stem diameter, relevant chlorophyll content, fresh weight/plant, dry weight/plant, essential oil %, and essential oil yield/plant by 21.00 and 9.94%, 58.70 and 40.00%, 20.69 and 15.83%, 68.83 and 58.28%, 48.70 and 56.16%, 45.71 and 35.53%, and 113.22 and 110.32% over the control in the two seasons, respectively. For total phenol and antioxidant activity, the most effective treatments were the ¾ NPK +1.5 g/L ZnO NPs and ½ NPK +2.0 g/L ZnO NPs, respectively. Simultaneously, essential oil composition (with their compound numbers identified (11–29 for control and ¾ NPK RD + 1.5 g/L ZnO NPs)) and the percentage of total compounds, monoterpene hydrocarbons, sesquiterpene hydrocarbons, and oxygenated hydrocarbons were varied among the used applications. The major observed compounds (>8%) estragole, methyl eugenol, linalool, cineole, and caryophyllene were found in different treatments. Thus, the findings of this study indicate the favorable utilization of ZnO NPs, SiO_2_ NPs, and DPPE in reducing the application of NPK, which may present a novel strategy and beneficial approach.

## 1. Introduction

*Ocimum basilicum* L., commonly known as sweet basil, is a native plant in tropical and subtropical regions and native to the family of Lamiaceae (Labiate family) [1]. Sweet basil is cultivated as medicinal or aromatic plants all over the world [2]. Basil herb is used in folk medicine as a remedy for many diseases, such as cancer, convulsion, diarrhea, epilepsy, gout, nausea, sore throat, toothaches, and bronchitis [3]. Basil herb is a source of essential oil containing biologically active constituents that possess antioxidant and antimicrobial properties [4,5]. Moreover, basil herb is utilized in the cosmetic industry [6]. In general, linalool methyl chavicol, citral, cinnamom, camphor, and methyl cinnamate are the important identified compounds in basil essential oil [7]. The aerial parts of basil herb contain up to 1% essential oil, and its rate is influenced by genetic factors, environmental factors, and soil fertility [1].

Nutrition is an important factor affecting the vegetative growth, essential oil productivity, and chemical composition of sweet basil. Nitrogen, phosphorus, and potassium are the major macroelements used in fertilizers of either volume or frequency. NPK are the major macronutrients applied in agriculture due to their essential roles in chemical, biochemical, and enthymemic activities, as well as metabolic processes in plant cells [8]. As a consequence of the decline in trace of microelements, such as Zn, Cu, B, Mn, and Fe, soils in numerous regions across the world have been rendered unreactive to NPK fertilizers. The unreactive soils can be attributed to the insufficient utilization of well-balanced fertilizers incorporating macro- or micro-nutrients and/or the pervasive depletion of micronutrients in continuously cultivated small Halder plots, resulting in the absence of these elements in the soil [9]. Chemical fertilizers supply plants with nutrients required for optimal growth and productivity. However, mineral fertilizers are intensively utilized to enhance plant production [10]. Furthermore, the intensive utilization of conventional fertilizers for long periods of time has caused serious environmental problems, including ground water pollution, water eutrophication, soil quality degradation, and air pollution [11]. Additionally, mineral fertilizers may affect heavy metal accumulation in the soil and plant systems. Thus, mineral fertilization could lead to water, soil, and air pollution [12]. The utilization of chemical fertilizers results in restricted efficacy in nutrient usage, while the limitations pertaining to the environment persist as a significant concern and obstacle towards achieving a rational sustainability in the field of agriculture [9,13]. Consequently, novel approaches in agriculture are being pursued that involve the application of environmentally friendly and safe products, encompassing a wide range of activities. These approaches are specifically designed to promote plant growth and alleviate the issues associated with increased environmental pollution caused by excessive use of chemical fertilizers.

In order to mitigate the risks associated with the extensive application of chemical fertilizers, it is possible to employ environmentally friendly and safe approaches to meet the nutritional and organic material needs of plants, such as nanotechnology and natural extracts. The utilization of nanoparticles (NPs) in agriculture has shown promising results in reducing the reliance on chemical fertilizers and enhancing plant growth across various plant species [14]. However, numerous studies have indicated that the effects of NPs on plants can vary, depending on factors such as their size and dosage, which can differ among different plant species [15,16]. NPs, which range in size from 1 to 100 mm in surface area, serve multiple functions and act as enhancers for plant growth, nutrition, and protection [17]. NPs offer a viable alternative for managing plant pests, fungi, and weeds. Furthermore, nanotechnology plays a crucial role in all stages of horticultural product production, storage, processing, packaging, and transportation, thereby revolutionizing the horticulture and food industries [18]. Nanotechnology has the potential to enhance food safety and improve sensory attributes, such as color, flavor, and texture, resulting in increased food reliability [19]. The quality and shelf life of horticultural crops can be improved through the application of nano-Si coatings on their surfaces in food packaging [20]. Packaging materials coated with Ag NPs can extend the shelf life of food products by reducing microbial activity, thus ensuring longer product viability [21]. Examples of organic and synthetic coating NPs, such as chitosan, Si, TiO_2_, and their derivative composites, have recently been used to coat fruits with short shelf lives, such as the Chinese bayberry, thereby extending their storage capabilities [22].

Among various NPs, ZnO NPs have been documented to be effective in promoting the growth of plant species [13,14]. There are some factors that affect the availability of zinc in soils and its harmful effect in plants, such as pH, soil physico-chemical characters, and the crop species/variety tolerance level [15]. Therefore, several studies have recommended the use of ZnO NPs as a foliar spray in order to limit the micro-nutrient reduction in plants [14,16]. ZnO NPs can be easily and directly absorbed in comparison to the soil utilization [17]. Zinc has vital roles in biomass and chlorophyll production, pollen function, RNA metabolism, and the formation of DNA [18]. On the other side, either a Zn deficiency or toxic levels of Zn have negative impacts on photosynthetic electron transport, photophosphorylation, and root membrane permeability that cause the electrolyte leakage of nutrients from the root [19]. Furthermore, Zn is an important micronutrient for plant growth and metabolism. It plays an essential role in several processes of metabolism, including cell wall metabolism, affect as structural elements in regulatory proteins, photosynthetic electron transport, mitochondrial respiration, and plant hormone biosynthesis, and as cofactors for different enzymes [20,21]. In addition, Zn increases the nutritional quality of food crops that are critical for human health [22,23].

Recently, silicon NPs have been employed as an important micronutrient in agriculture, mainly in arid environments, to hold water and bind other nutrients, thus leading to increased cell strength [24]. Additionally, the application of Si enhances plant photosynthesis, chlorophyll content, and product quality [17]. It alleviates the negative impacts of diseases and abiotic stresses on plants [25,26,27,28]. According to Ahanger et al. [29] the utilization of silica (Si) resulted in an increase in the concentration of secondary metabolites in various plant species, serving as a defensive mechanism against multiple stressors. Additionally, the utilization of Si nanoparticles (NPs) has been shown to enhance stomatal conductance, the electron transfer rate, and phytochemical processes. Furthermore, it is important to note that there is currently no evidence suggesting that silica poses any ecotoxicological risks towards birds, fish, invertebrates, microorganisms, or plants.

Date palm (*Phoenix dactylifera* L.; Palmaceae) pollen grains are considered as one of the most effective and are commonly used in the Middle East, especially in Egypt. Pollen grains are gathered from the male trees of date palm. Date palm extract has various constituents, like enzymes (that are analyzed via electrophoresis [30]), sterols, triterpenes, saponins, proteins, vitamins A, C, and E, elements, such as N, B, Zn, Se, Fe, Mo, Cu, and Mn, carbohydrates, glycosides, rich with many amino acids, and 13 fatty acids, such as palmitic acid (34.45%), phenyl ethanol (8.75%), volatile oils, antioxidants, total phenolics, and flavonoids, as well as different steroids, such as brassinosteroids [31,32]. All these compounds are important because they affect the physiological, enzymatic, and biochemical processes in plants. Consequently, it takes into account plant growth and secondary metabolism in plants. However, the influence of the combined use of NPK, date palm pollen extract (DPPE), ZnO NPs, and SiO_2_ NPs on vegetative growth, essential oil (EO) productivity, and chemical composition has not been determined in any plant species. Therefore, the aim of the present field experiment was to determine the effects of NPK fertilizers, NPs of ZnO and SiO_2_, and DPPE on the growth, essential oil productivity, and chemical composition of *O. basilicum* and to assess their utilization as a safe and cheap alternative fertilization source to NPK fertilizers in order to reduce environmental pollution and ensure the formation of safe products.

## 2. Results

### 2.1. Vegetative Growth Parameters

Data recorded for the different growth traits (plant height, branch number/plant, main stem diameter, relative chlorophyll content, and fresh and dry weights of aerial parts) are presented in Table 1 and Table 2 as the averages of the two cuts of each season.

The maximum significant plant height (Table 1) that resulted from the ½ NPK RD + 2.0 g/L ZnO NPs was recorded as 24.66 and 27.07% over the control in both seasons, consecutively. The maximum significant branch number value (Table 1) was recorded for plants that received the ½ NPK RD + 20 g/L DPPE + 150 mg/L SiO_2_ NPs and ½ NPK RD + 20 g/L DPPE + 2.0 g/L ZnO NPs treatments, which resulted in 21.00, 21.00, 3.87, and 9.64% increases in branch number over the control plants (NPK RD) in both seasons. However, the differences among these treatments were not significant (*p* ≤ 0.05), especially for the 2022 season. Conversely, some treatments decreased shoot branching in comparison to the control in both seasons, for instance, the ¾ NPK RD plus either 10 g/L DPPE or 150 mg/L SiO_2_ NPs.

Concerning the main stem diameter, there were no significant differences among the applied treatments, especially for the 2022 season (Table 1). The main stem diameter was significantly augmented by applying the ½ NPK RD + 20 g/L DPPE + 2.0 g/L ZnO NPs relative to the other treatments with some exceptions. Also, such a treatment had achieved 58.70 and 40.00% increments in the main stem diameter over the control during the experimental seasons.

All fertilizer supplements significantly improved relative chlorophyll content (RCC) compared to the NPK RD, with some exceptions, where the increment was non-significant (Table 1). The plants that received the ½ NPK RD combined with either 2.0 g/L ZnO NPs or 20 g/L DPPE + 2.0 g/L ZnO NPs had the significantly maximum SPAD values compared to the control. Such treatments increased SPAD values by 18.99 and 20.69% consecutively during the 2021 season, while during the 2022 season, the increments were 18.33 and 15.83%, respectively.

As for the fresh and dry weights of aerial parts, the data in Table 2 affirm the positive behavior of the ZnO and SiO_2_ NPs and DPPE in reducing mineral NPK fertilizer utilization. Also, it was obvious that the differences among the used treatments were significant (*p* ≤ 0.05) in most of the cases. The highest values of the fresh and dry weights of aerial parts resulted from the fertilized plants with the ½ NPK RD + 20 g/L DPPE + 2.0 g/L ZnO NPs in both of the seasons. Furthermore, supplying the plants with this application improved the fresh weights of aerial parts by 68.83 and 58.28% and the dry weights of aerial parts by 48.70 and 56.16% in comparison to the control plants over the two seasons, respectively. Also, all the used applications significantly resulted in increases in aerial parts’ fresh and dry weights over the control.

### 2.2. Essential Oil Productivity

**Figure 1:**
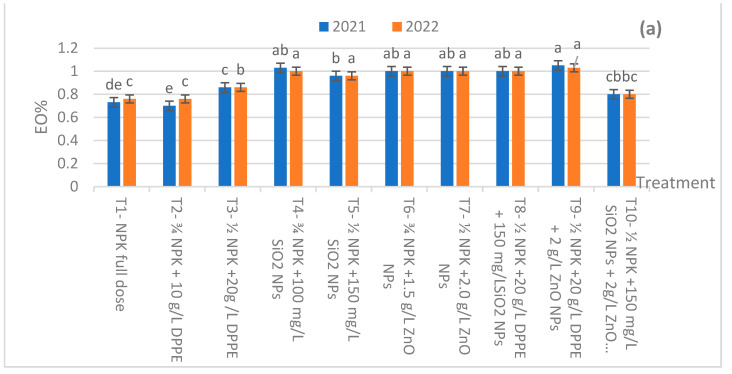

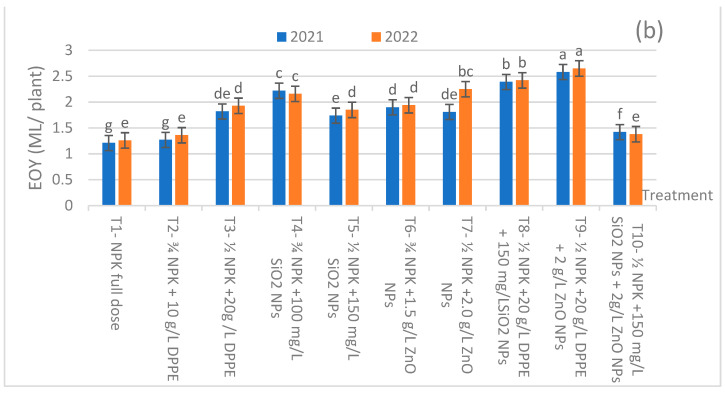
(**a**) Essential oil % as affected by the used applications (average: two cuts) during the 2021 and 2022 seasons. (**b**) Essential oil yield/plant as affected by the used applications (average: two cuts) during the 2021 and 2022 seasons. Means with the same letters within the figure are not significantly different (*p* ≤ 0.05) according to DMRT.

The data illustrated in Figure 1a,b show the effect of different fertilization treatments (as an average of two cuts) on basil essential oil percentage (EO %) and essential oil yield (EOY)/plant. Compared to the control, the used fertilization treatments had significantly pronounced increments in either the EO % or EOY/plant in both seasons, with some exceptions. Simultaneously, regardless of the control, most fertilizer application plants had close EO % values. At the same time, the differences in the means of EO % or EOY/plant among most treatments were non-significant, especially for the EO % of the second season. Clearly, the plants that received the ½ NPK RD + 20 g/L DPPE + 2.0 g/L ZnO NPs in the two seasons had the maximum EO % at their aerial herbs. Such plants had EO % values of 1.02 and 1.03%, compared to 0.70 and 0.76% for the control plants in the two seasons in succession. Simultaneously, the maximum EOY was 2.58 and 2.65 mL/plant for the fertilized plants with the ½ NPK RD + 20 g/LDPPE + 2.0 g/L ZnO NPs in the two seasons, consecutively. On the other side, the minimum EOY was recorded for the control plants, which resulted in 1.21 and 1.26 mL/plant in the two seasons, respectively.

### 2.3. Essential Oil Composition

The analysis of the EO samples revealed that the treatments used in this study had pronounced impacts on the EO compounds (Table 3). A total of 11 components were found in the control. The identified compounds in the other treatments ranged from 12 components in the plant’s EO that were subjected to T5 and T7 to 29 compounds in the plants that were related to T6. At the same time, a total of 44 compounds were noticed in basil EO distribution among the used treatments. Though, some compounds were observed in all fertilization treatments such as cineole, estragole, methyleugenal, caryophyllene, cis-a-bergamatene, and germacrene D. The percentage of such compounds differed according to the treatments. The results revealed that total compounds ranged from 92.84% for T6 to 99.99% for T4, T7, and T10. Also, monoterpene hydrocarbons ranged from zero % in the control to 1.63% for T5, and sesquiterpene hydrocarbons ranged from 11.29% for T4 to 25.10% for T8, while the oxygenated hydrocarbons (which are compounds that contain oxygen) ranged from 66.99% in T6 to 88.26% in T4. The highest major compounds % of different treatments (>4%) were stragol (72.12%, T4), methyl eugenol (47.89%, T1), linalool (20.44, T6), cineole (11.47%, T5), caryophyllene (8.59%, T7), 1H. benzocycloheptene, 2,4a,5,6,7,8,9,9a-octahydro-3,5,5- trim ethylene-, (4as-cis) (6.30%, T2), CYCLOHEXENE, 4-(1,5-DIME7HYL-1,4-HEXADI- ENYL)-1-METHYL-(5.55%, T8), cis-a-bergamotene (5.85%, T6), and germacrene D (4.68, T2). Compared to the control, it was noted from the data that all the used treatments contained a ¾ or ½ NPK dose confirmed with ZnO NPs, SiO_2_ NPs, or DPPE, which increased the number of EO compounds, total compounds (except for T6), monoterpene hydrocarbons, and sesquiterpene hydrocarbons (except for T3, T4, and T5) and decreased the oxygenated hydrocarbons (except for T3, T4, and T5). 

### 2.4. Total Phenolic Compounds (TPCs) and Antioxidant Activity (AOA)

Impacts of the used fertilization treatments on the TPC and AOA of leaf basil extracts are illustrated in Table 4. The data clearly show that the basil foliar spray with ZnO NPs, SiO_2_ NP, and DPPE combined with the ¾ or ½ NPK RD significantly boosted the TPCs relative to T1 (control), except for T2 and T3, which significantly decreased the level of TPCs than T1. Among the treatments, the highest significant TPC value was found in the plant leaves that received T6, which contained 12.22 mg GAE/g dry weight (D.W). The following treatment was T7, which resulted in 11.25 mg GAE/g D.W; then, T10 gave 11.20 mg GAE/g D.W. It was noticed from the data of TPCs that the differences among all the used applications reached a significant level.

Concerning AOA, the used applications that contained ZnO NPs, SiO_2_ NPs, and DPPE combined with the ¾ or ½ NPK RD achieved AOA values that were nearly all higher than the T1 (control) value. T7 enhanced AOA (0.03321 micromole Trolox equivalent (µMTE)/10 g D.W) over the other ones. AOA ranged from 0.02542 µMTE to 0.03321 µMTE/10 g D.W for T1 and T7, respectively.

## 3. Discussion

Fertilization is considered one of several important factors affecting the growth, yield, and chemical composition of plants. Our current results indicated the positive impact of NPs of ZnO and SiO_2_, or DPPE as a foliar spray in order to reduce the intensive NPK application. All treatments containing the ¾ or ½ NPK RD combined with different concentrations of NPs of ZnO and SiO_2_ or DPPE significantly improved sweet basil vegetative traits relative to the NPK RD, with some exceptions observed, particularly in the branch number trait. According to Paramo et al. [33], the role of nanotechnology in agriculture includes the abiotic stress tolerance mechanism of many plant species. ZnO NPs act as a source of Zn that can be accumulated in the leaves during foliar spray, and may act as active Zn sources that can be utilized in plant metabolism activities [34]. ZnO NPs are supplied as nano-nutrients and are considered a more efficient and slow-release source of Zn than traditional fertilizers [35,36]. Also, the positive impact of ZnO NPs on basil growth parameters may be due to its important role in several functions in plant cells, including physiological, chemical, and biochemical processes [20,21]. Our findings confirm a previous study on *Alyssum desertorum*, *Barago officinalis*, *Calendula officinalis,* and *Thymus vulgaris*, in which Zn and Fe positively affected the agronomic characters of these species [37]. Also, the physiological traits of *Lavandula officinalis* were significantly affected by zinc utilization under salinity conditions [38]. ZnO NPs at 6 mg/L resulted in the heaviest significant basil shoot fresh and dry weights in comparison to 0, 2, and 4 mg/L of nano Zn or 2, 4, and 6 mg/L of either nano Fe or nano K [39]. Likewise, a parallel relationship between the basil growth traits and NPK NPs (0, 2.5, 5.0, 7.5, 10.0, 12.5, and 15.0 mg/10 L) rate was reported [40]. The authors found that a moderate level of NPK NPs was sufficient for enhancing the SPAD trait. Previous studies on *Origanum majorana* [41], *Rosmarinus officinalis* [42], and *Mentha piperita* [43] concluded that the foliar application of ZnO NPs improved the morphological parameters of these plant species relative to untreated plants. From their studies, the impact of ZnO NPs is dependent on its concentration, plant species, and application stage. Similarly, both zinc chelate and nano zinc had raised chlorophyll indices in *Borago officinalis* relative to the control [44]. Pirzad and Barin [45] mentioned that foliar spraying of 4 g/L zinc improved the biological yields of *Pimpinella anisum*. For rosemary plants, it was found that Zn NPs at 3 mg/L had a positive impact on the above-ground traits and total chlorophyll [42]. In our study, the application of ZnO NPs alone and the ¾ or ½ NPK RD or combined with the ½ NPK RD + either 20 g/L DPPE or 150 mg/L of SiO_2_ NPs did not show any toxic symptoms on the basil plant. In contrast to our results, it was observed that ZnO NPs at 500 mg/L caused toxicity in *Arabidopsis thaliana* growth [46].

Recent studies highlighted the positive behavior of SiO_2_ NPs on basil morphological traits, consequently reducing the utilized amount of NPK. The treatments, including SiO_2_ NPs, significantly enhanced the basil growth traits across the two seasons, with some exceptions relative to the control. Similarly, Si is beneficial for plant growth as it improves the photosynthetic rate and helps fight against various biotic and abiotic stresses [26,27,47]. Therefore, the increased growth potential observed in the aerial parts of basil plants could be attributed to the advantageous impact of SiO_2_ NPs on plant metabolism. Interestingly, no harmful impacts of SiO_2_ NPs on the basil plant were noticed under the concentrations used. The positive effects of SiO_2_ NPs on SPAD traits in our study conditions are in agreement with the studies mentioned before, where SiO_2_ enhanced plant growth via increasing photosynthetic level, photosystem 11 activity, stomal conductance, electron transport rate, and photochemical quenching [48,49]. *Arabidopsis thaliana* seedling growth was promoted by 10 mg/L of SiO_2_ NPs, and chlorophyll content was improved even at higher rates (0–500 mg/L) [46]. *Changlrai larch* seedling growth was increased by foliar spraying of SiO_2_ NPs, mainly by enhancing root growth and chlorophyll synthesis [50]. Also, Si increased the growth biomass of plants due to its effects on reducing heavy metal uptake or increasing essential nutrient uptake and improving photosynthetic pigments [51,52]. The Si foliar spray impacted the green color index of orchids [53]. Likewise, on sage plants, it has been reported that the foliar spray of Si or nitric oxide, and particularly their combinations, restored chlorophyll in leaves that improved photosynthesis and consequently enhanced the shoot biomass of Cu-stressed plants [54]. Our results resemble those of Al-Saidi et al. [55]. The authors showed that treating fenugreek plants with 200 kg/ha NPK (20:20:20) + 2 g/L nanoelements (B 0.5%; Mo 0.5%, Cu 0.5%, Zn 1.5%, Mg 1.5%, and Fe 8%) + nano seaweed extract at 2 g/L resulted in the highest values of vegetative growth traits relative to the control.

The recent investigation placed a significant emphasis on the pronounced positive impact of DPPE on basil growth traits when the NPK supply was decreased. Conversely, the application of DPPE at the utilized concentrations enhanced basil growth traits in comparison to the NPK RD, with a few exceptions, particularly in shoot branching during the two seasons. The available information on the use of DPPE as a natural extract in agriculture is extremely limited, hence forming the rationale behind our study. Overall, the enhancement of basil growth parameters through the utilization of DPPE can be attributed to its aforementioned constituents, which play a crucial role in plant growth. Our findings are in accordance with those of Abou-Sreea and Yassen [56], who demonstrated that 20 g/L DPPE was the most effective for promoting the bird of paradise growth traits in comparison to untreated plants and 5, 10, 15, and 25 g/L DPPE. The addition of 200 mg/L of water pollen extract to tissue culture medium enhanced the shoot number, length, fresh weights, and dry weights of the banana plant [42]. In general, many authors documented that the improving effect of DPPE on plant growth may be referred to that pollens contain auxins and cytokinins [57,58,59].

We can deduct from our results that NPs of ZnO and SiO_2_ or DPPE alone or in combination with NPK at the ½ or ¾ RD positively affected basil EO % and EOY, consequently reducing the utilization of the NPK fertilizer. On the basis of the previous literature, it can be hypothesized that many factors have an impact on EOY. These factors include climatic conditions, such as rainfall and water, light, temperature, relative humidity, air, and wind, as well as environmental factors, like salinity and geographical origin [60]. In addition, agricultural practices, including fertilization and controlling weeds, and insects play a significant role. According to a study by the authors of [51], a non-linear relationship exists between the rate of NPK NPs and basil EO (expressed as a percentage by weight). It appears that a balance is achieved between macro- and micro-nutrients when NPs of ZnO and SiO_2_ or DPPE are added, as these substances contain various compounds and elements. This balance may enhance all cellular processes in plants, including secondary metabolism, which, in turn, leads to increased EO synthesis. Another study by the authors of [61] found that concentrations of Ca++ and K+ have a strong influence on EO yield. Furthermore, Zn is involved in the synthesis of carbohydrates, protein metabolism, and the regulation of auxins, all of which contribute to EO content [62].

It has been demonstrated that 400 mg/L of Zn as a foliar spray increased the EO % of coriander [63]. A significant influence on the EO % of basil was observed when 2, 4, and 6 mg/L of F, K, and Zn nano-chelates were used [64]. Furthermore, some studies showed the enhancing impact of Zn NPs alone or combined with other nutrients on the EO productivity of many species, such as *Pimpinella anisum* [45], sweet marjoram [65], *Brassica nigra* [66], and *Mentha piperita* [67]. Likewise, it has been mentioned that in drought conditions, soil application of Si improved the EO % of *Ocimum basilicum* [38]. It has been documented that Si enhanced the content of secondary metabolites in many plant species as an attack response versus different stresses [68]. Under drought stress, rose EO content was increased with Si foliar application [69]. Additionally, it was found that Si at 1 mM foliar application resulted in an increment in the EO content of sage leaves under Cu stress [54]. Elicitation with DPPE improved the EO % and EOY in basil herbs under the recent study conditions. To our knowledge, there are no available studies about the use of pollen grains on aromatic plants. Therefore, we deduce that improving EO synthesis in basil may be due to several DPPE compounds that have positive effects on physiological, biochemical, and chemical processes and enzymatic activities, which turn into EO synthesis.

The obtained results of the basil EO analysis may be due to the important role of combinations among the ¾ or ½ NPK RDs and the used level of nano ZnO, nano SiO_2_, and DPPE in activating the metabolic processes and enzymatic activity. K is active in several important processes of metabolism in plants that improve transportation in the phloem, osmotic balance, and photosynthesis [54]. N is an essential constituent of secondary metabolism. P is important for the transfer of energy and carbohydrates in leaf cells [8]. According to [70], NPs stimulate secondary metabolites, such as terpenoid compounds. NPs have enhanced secondary metabolism, biosynthesis, and gene expression [71]. Also, Zn is a vital element for plant hormone biosynthesis and functions as cofactors for many enzymes [20] in RNA metabolism, DNA formation, carbohydrate synthesis, and saccharide metabolism [18,72].

Secondary metabolism content and phytochemical processes in several plants were improved in response to Si NP application [29,68]. Additionally, various constituents of DPPE, like enzymes, proteins, vitamins A, C, and E, macro- and micro-elements, carbohydrates, etc., are important in secondary metabolism [31,32]. Also, ref. [39] reported that nano-chelate fertilizers (F, K, and Zn) at 2–6 mg/L affected the basil EO compounds (percentage and amount) except for methyl chavicol. The same authors added that 28 compounds in basil EO were identified in total; 15 compounds were obtained from the Zn nano-chelates treatment, 10 compounds from iron, and 13 compounds from the K treatment. They added that Zn nano-chelates, at different levels, enhanced EO composition; linalool (42.2–26.2), 1,8-cineal (1.9–2.2), eugenol (5.9–6.91), &-terpinol (7.3–5.4), epi-&-muurolol (2.5–3.9), E-B-ocimene (2.2–3.8), and carvone (18.8–20.84) were the main compounds. The authors of Ref. [73] on *Salvia officinalis*, when they treated it with NPK at 100, 75, and 50% alone or combined by biofertilizers, found that EO compounds (number and total %) and the percentage of oxygenated hydrocarbons, monoterpenes, and sesquiterpenes differed under their different fertilization treatments. Indeed, this study, along with the other ones, suggested that the major compounds of basil EO are varied due to the climatic and nutritional conditions. Accordingly, ref. [74] concluded that the same compounds % of basil EO grown in different climatic conditions of three governorates in Egypt were different.

Concerning the phenolic compounds, which have a vital role among plant constituents due to their scavenging potential on free radicals, refer to their hydroxyl groups. Thus, the plant’s phenolic content may be directly due to its antioxidant action [61]. It has been demonstrated that the plant extract’s antioxidant activity has showed a consanguinity between total phenolic contents and antioxidant activity [62,63]. Moreover, a previous study [66] reported that phenolics are essential phytochemical constituents with significant antioxidant ability. This is further supported by findings on rosemary [75], sunflower [76], and lavender plants [77], concluding that there is a positive relation between the total phenolic content and antioxidant activity of plants. Our results may be attributed to the positive effects of Zn NPs, Si NPs, and DPPE (that contains several macro- and micro-elements and various biochemical compounds) on the TPC and AOA of the plant extract. Accordingly, it was revealed that the beneficial impacts on AOA potency may refer to the assumption of the role of Zn on phenol biosynthesis, membrane integrity, maintaining the high contents of ROS scavenging molecules, structural molecule guarding, holding sulfhydryl groups, and the prevention of unwanted behaviors between Fe and other chemical groups [78]. Thus, Zn has an essential role in maintaining cell membranes against stressful conditions. Ref. [42] mentioned that Zn NPs at 3 mg/L increased TPC content in rosemary in comparison to untreated plants. Similarly, ref. [14] found that ZnO NPs at 25, 50, and 100 mg/L boosted up the antioxidant system in wheat.

Si improved phenolic compound productivity in plants by increasing the activity of enzymes involved in the phenylpropanoid pathway, like phenylalanine ammonia-lyase (PAL) [29]. Previous research revealed that SiO_2_ NPs can increase the TPCs and flavonoid contents, rosmarinus acid, and xantomicrol, and raise AOA in *Dracocephalum kotschyi* Bios hairy roots by upregulating rosmarinic acid synthase and (PAL) expression genes [79]. Additionally [67], it has been reported that treated sage plants with 1 mM Si or 200 mM Si NPs enhanced TP and DPPH scavenging activity. Similarly, it was found that the utilization of 75% NPK + biofertilizer (*A. chroococcum*, *B. megaterium var. phosphaticum*, and *B. cereus*) on sage plants caused an increment in TP compounds and AOA in comparison to NPK at 50 and 100% [73].

## 4. Materials and Methods

A field experiment was carried out in a private farm at Meet Yazid village, Kom Hamada city, El-Behera Governorate, which is located 125.4 km away from Cairo, Egypt, during two successive seasons (2021 and 2022) to evaluate the response of sweet basil (*Ocimum basilicum* L.) to the partial substitution of the NPK fertilizer with the foliar spray with NPs of zinc oxide (ZnO) and silica oxide (SiO_2_) and date palm pollen extract.

### 4.1. Soil Analysis of the Experimental Site

Soil samples of 0 to 30 cm depth were taken from five spots from the area of the experimental site (100 g/spot) during the season of study before starting the experiment, and the samples were carefully in one sample to ascertain the physiochemical parameters of the soil that was used. A mortar and pestle were used to ground the soil air-dried samples, which were then passed in a stainless steel test sieve into fractions <2 mm [80] clayey sand soil (clay 65.33%; sand 29.40%; and silt 5.27%) was the soil texture. The particle size distribution was analyzed using the hydrometer method [81]. The soil pH, EC, organic matter (O.M) %, sodium adsorption ratio (SAR), and exchangeable sodium percentage (ESP) % were 7.80, 2.22, 2.31, 2.77, and 2.70%, respectively. Soil-available NPK were 815, 30.5, and 56.7 mg/kg. The soil soluble cations Na^+^, K^+^, Mg^++^, Ca^++^, and Zn^++^ were 7.1, 0.9, 4.3, 9.8, and 0.81 meq, respectively. The land soil-soluble anions CO_3_^−−^, Cl^−^, HCO_3_^−^, and SO_4_^2−^ were 0.0, 7.0, 3.9, and 11.3 meq/L, respectively. For this determination, the following soil chemical parameters were employed: 20 g dried soil/100 mL distilled water (1:5), and were left for 24 h; then, the extract was filtered. The measurements in the extract filters were carried out as follows: an EC meter (MI 170, SZ egged, Hungary, Italy) was used to determine soil EC [82]. Jackson’s method [82] was also applied for the estimation of Ca^++^, Mg^++^, and Cl^−^. Total carbonate and organic matter were quantified using the method of [83]. The micro Kjeldahl method was used for estimating the level of available N [84]. Available P was estimated using the method outlined by the authors of [85]. Na^+^ and K^+^ were determined with the PSC7 flame photometer (JENEWY, Staffordshire, UK), and SAR and ESP were also estimated [86]. Zn^++^ was quantified with the atomic absorption spectrophotometer (AAS) [87]. Soil pH was measured in soil suspension (1:2.5, soil: distilled water) after 30 min using a pH meter (JENEWAY3510, Staffordshire, UK) [86].

### 4.2. Plant Preparation

Basil seeds were provided by the Medicinal and Aromatic Plants Depart. Hort. Res. Inst. Agric Res. Center, Ministry of Agric., Egypt. The seeds were sterilized for 5 min with 5% commercial sodium hypochlorite and then rinsed with distilled water. Seeds were sown on 1 February 2021 and 2022 in plastic seedling trays that were filled with peat moss; then, they were placed in an air conditioner plastic greenhouse adjusted 25 ± 2 °C, 40–50% relative humidity, and a photoperiod 16 h light/8 h dark with a light intensity of 300 m mol m^−2^ s^−1^. The plastic trays were irrigated manually using 10-L watering cans with Nile water (comprising a pH of 7.33 and an EC of 0.35 dsm^−1^) when they were needed. After the germination procedure, the seedlings (12 cm in height) were transferred to the field on 10th March 2021 and 2022 in an experimental plot (unit) (2 × 2 m of each) in hills 30 cm apart between plants and 60 cm between rows (with each row containing 7 hills). Each plot contains 4 rows cultivated with 28 plants. The irrigation system in the experimental farm is flooded with Nile water. 

### 4.3. Fertilizer Types

#### 4.3.1. NPK Fertilizers

Ammonium sulfate (20.5% N), calcium super phosphate (15.5% P_3_O_5_), and potassium sulfate (48% K_2_O) at rates of 240, 150, and 75 kg/fed., respectively, were applied as the recommended dose (following the Ministry of Agriculture, Egypt), where calcium super phosphate was added as one dose when the soil was prepared. Meanwhile, ammonium sulfate and potassium sulfate were divided into 4 equal doses, and they were applied as soil drenching. The first dose was added after one month from transplanting on 9th April and the second one after two months from transplanting on 9 May; the third dose was utilized 15 days after the first cut; finally, the fourth dose was added 45 days after the first cut during the 2021 and 2022 seasons. 

#### 4.3.2. Nanoparticles

Zinc oxide nanoparticles (ZnO NPs) at 1.5 and 2 g/L, after [45,88], and silicon nanoparticles (SiO_2_ NPs) at 100 and 150 mg/L, after [89], were used as foliar sprays. A scanning electron microscope (SEM) (Quanta FEG250, FEI, Waltham, MA, USA) was employed for observations. An accelerating voltage of 20 kV was also utilized. A detector for LFD spot sizes of 3.5 was also used, and a low vacuum mode was maintained. EDX ThermoScientific Dry/wet EDX. The presence of functional groups was characterized via ATR–FTIR spectroscopy, THERMO NICLOT, Oxford, UK. X-ray diffraction measurements were carried out with a Bruker D2 phaser, 2nd gen, Bremen, Germany.

##### Synthesis of NPs of ZnO and SiO_2_

**Figure 2:**
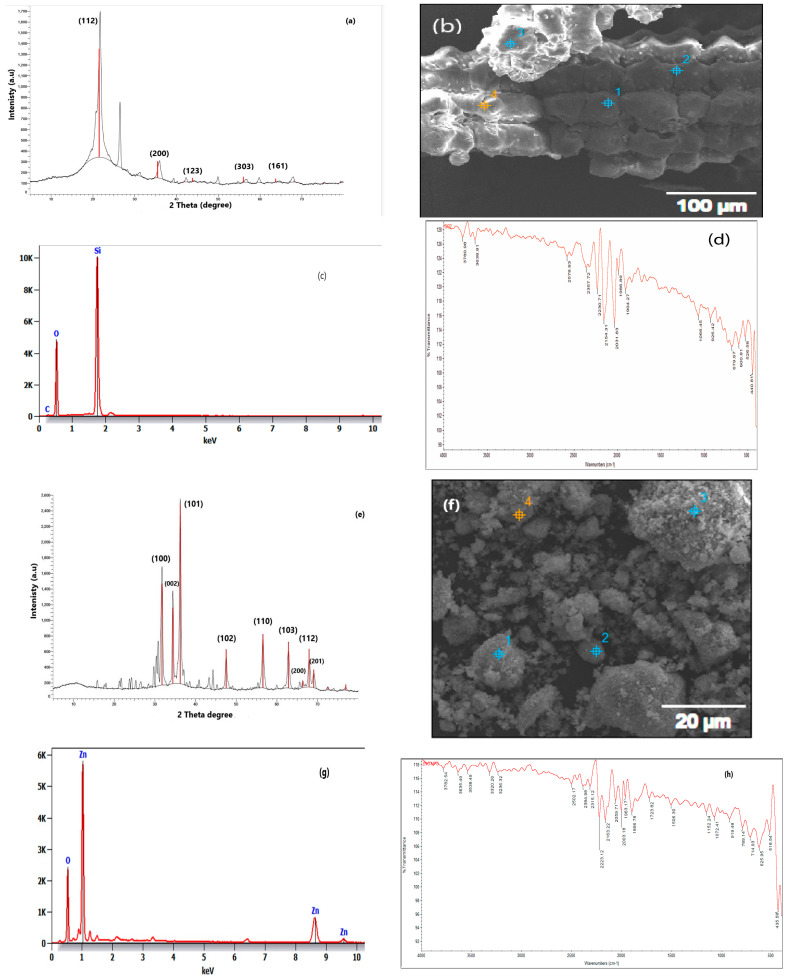
(**a**) XRD of SiO_2_ NPs; (**b**) SEM of SiO_2_ NPs; (**c**) ADX of SiO_2_ NPs; (**d**) FTIR of SiO_2_ NPs; (**e**) XRD of ZnO NPs; (**f**) SEM of ZnO NPs; (**g**) ADX of ZnO NPs; and (**h**) FTIR of ZnO NPs. 1—Fourier-transform infrared (FTIR) absorption spectroscopy was used to evaluate the chemical bonding nature of the nanoparticles. 2—Scanning electron microscopy (SEM) was used to examine the morphology and elemental composition of the powder as it was removed [92]. 3—X-ray diffraction (XRD) is a technique for characterizing crystalline materials. It provides information on structures, phases, preferred crystal orientations (texture), and other structural parameters, such as average grain size, crystallinity, strain, and crystal defects [93]. 4—Energy-dispersive X-ray spectroscopy (EDX) is a technique of elemental analysis associated with electron microscopy based on the generation of characteristic X-rays that reveals the presence of elements present in the specimens [94].

The ZnO NPs were synthesized using the coprecipitation method, as described by [90]. Briefly, 100 mL of 1 mM Zn(CH_3_COO)_2_. 2H_2_O was dropwise mixed with 50 mL of 2 m NaOH with constant stirring for 2 h. The white precipitate was collected via centrifugation (model 58 10r, Eppendorf corporation, Hamburg, Germany) at 9508 for 5 min at room temperature (25 ± 2 °C) and washed thrice with distilled water to remove impurities. The ZnO NPs were dried overnight in a drying incubator (Thomas Scientific, Swedesboro, NJ, USA) at 60 °C. The method of [91] was used for the preparation of SiO_2_ NPs via the sol–gel process. For the preparation of SiO_2_ NPs via the sol–gel method, 35 mL of H_2_O was mixed with 65 mL of absolute alcohol for 5 min under mechanical stirring. After that, 25 mL of tetraethyl orthosilicate (TEOS) was added dropwise to the previous ethanol/water solution and kept under mechanical stirring for 60 min at room temperature. To this end, an ammonia solution was added dropwise until the complete formation of gel was ascertained. Thus, it was noted that the solution was converted into a gel (sol–gel process). The formed gel was submitted to ultra-centrifugation for 2 h at 7000 rpm. Finally, the precipitated wet gel was collected and washed three times with distilled water in order to remove the undesired/unreacted compound (TEOS). The wet gel was subjected again to ultracentrifugation. At the end, the obtained gel was left for calcination at 700 °C for 5–7 h. An Empyrean PANalytical X-ray diffractometer with Bragg–Brentano geometry using Cu Ka radiation (R = 1.54 A) was used to record the powder patterns of the NPs of ZnO and SiO_2_. The step scan covered the angular range of 20–80 with a step of 0.02. The crystallite size was determined using the Scherrer equation, D = Kh/B cos B, where D is the crystallite size, K is a constant (0.94), h is the wave length of the X-ray radiation, B is the line width at half the maximum intensity of the peak, and QB is the angle of diffraction. The specification, physico-chemical properties, and acute toxicity of ZnO NPs and SiO_2_ NPs are shown in Table 5. Figure 2a–d show SEM, FTIR, XRD, EDX of SiO_2_ NPs and Figure 2e–h show SEM, FTIR, XRD, EDX of ZnO NPS.

The NPs of ZnO and SiO_2_ were sprayed thrice. The first and second sprays were conducted at 35 and 65 days following the transplanting of the basil seedlings, respectively, and the third one was carried out 25 days after the first cut.

#### 4.3.3. Date Palm Pollen Extract (DPPE)

Pollen of Egyptian date palm (*Phoenix dactylifera* L.) were harvested at the end of the March 2021 and 2022 seasons at the beginning of opening covers of the male species from Rashed city, Elbehyra Governorate, Egypt. The extract of pollen was prepared according to the authors of [95], with some modifications, as follows: to prepare the water pollen extract, 0.1 g of pollen grains was added to 10.0 mL of distilled water. After one hour, the mixture was sonicated using a VCX 750 ultrasonic probe (SONICS & MATERIALS, INC., Newtown, CT, USA) (frequency of 6 kHz), cut for 30 s, and then centrifuged (Sigma 3–18 KS, SIGMA Laborzentrifugen GmbH, Osterode am Harz, Germany) at 5000 rpm for 10 m in at a temperature of 20 °C. The resulting supernatant was used as the water pollen extract in all the experiments. Then, the volume was completed with water to obtain the used concentrations (10 and 20 g/L) [56]. The tested bio-stimulant was applied as a foliar spray thrice. The 1st and 2nd sprays were carried out after transplanting at 32 and 62 days, respectively, and the third one was conducted 22 days after the first cut. For the NPs and DPPE, tween 80 was applied as a sticking agent in each spray. The spraying was carried out with a hand sprayer in the morning. The plants were sprayed till the spray ran off. 

### 4.4. The Fertilization Treatments

Ten fertilization treatments in this study were conducted as follows: T1—NPK RD (recommended dose) as a control, and the control plants were sprayed with tap water, T2—¾ NPK RD + 10 g/L date palm pollen extract (DPPE), T3—½ NPK RD + 20 g/L DPPE, T4—¾ NPK RD + 100 mg/L SiO_2_ NPs, T5—½ NPK dose + 150 mg/LSiO_2_ NPs, T6—¾ NPK RD + 1.5 g/L ZnO NPs, T7—½ NPK RD + 2.0 g/L ZnO NPs, T8—½ NPK RD + 20 g/L DPPE + 150 mg/L SiO_2_ NPS, T9—½ NPK RD + 20 g/L DPPE + 2.0 g/L ZnO NPs, and T10—½ NPK RD + 150 mg/L SiO_2_ NPs + 2.0 g/L ZnO NPs, where ZnO and SiO_2_ are in the form of NPs, and their solutions were prepared with distilled water. Different agricultural practices (controlling weeds and application pesticides, etc.) were applied during the growing season. 

### 4.5. Experimental Layout

The investigation was subjected to a randomized complete block design with three replicates. Each replicate contained 10 treatments [96], and each treatment included 3 plots (experimental units) in one way.

#### 4.5.1. Vegetative Growth Traits

The first and second cuts of basil were carried out on 12 June and 10 August during each season (2021 and 2022), respectively, where 3 plants from each plot (experimental unit) were selected randomly, and these traits were estimated (as an average of 2 cuts of each season) as follows: plant height (cm) was measured from the soil surface to the top of the plant, branch number/plant, main stem diameter (cm) at 5 cm from the soil surface, fresh weight and constant air dry weight of aerial parts/plant (g) (after the plants were cut, they were processed immediately in the laboratory. They were weighed fresh and then placed on wood tables to dry for the constant air dry weight after 7 days, and a Minolta SPAD chlorophyll meter model-502 was used to measure relative chlorophyll content (RCC) as SPAD units for the fifth leaf from the branch top [97].

#### 4.5.2. Essential Oil Percentage and Yield

Air-dried basil samples of aerial parts (25 g/sample) were subjected to hydrodistillation with 0.5 L of sterile water for 3 h using a Clevenger-type apparatus. The obtained essential oil (EO) was dried over anhydrous sodium sulfate and stored at 4 °C for further use [98], where:

$$\mathrm{EO}\;\%=\frac{\mathrm{oil}\;\mathrm{volume}\;\mathrm{in}\;\mathrm{graduated}\;\mathrm{tub}\;}{\mathrm{weight}\;\mathrm{of}\;\mathrm{plant}\;\mathrm{sample}\;}\times 100\;\;\mathrm{EO}\;\mathrm{yield}/\mathrm{plant}\left(\mathrm{mL}\right)=\mathrm{EO}\;\%\;\times \;\mathrm{weight}\;\mathrm{of}\;\mathrm{air}\;\mathrm{dried}\;\mathrm{aerial}\;\mathrm{parts}/\mathrm{plant}.$$

where EO % and EO yield/plant were calculated as an average of the two cuts of each season. 

#### 4.5.3. Gas Chromatography/Mass Spectrometry Analysis of Oil

GC–MS analysis was carried out for EO at the 2nd cut during the 2nd season; the sample was taken and filtered so that it did not affect the column, and then 1 microliter of the sample was taken and injected into an Agilent 6890 N gas chromatograph equipped with a DB-5 MS capillary column (30 m × 250 μm × 0.25 μm, Agilent Technologies, Santa Clara, CA, USA) and coupled with a 5975 B mass-selective detector spectrometer from the same company. The front inlet was kept at 250 °C in split mode. The temperature program was as follows: the initial column temperature was 60 °C, held for 2 min, and then programmed to 120 °C at a rate of 6 °C per minute and held for 2 min; finally, it was programmed to 230 °C at a rate of 4 °C per minute, held at 5 min. The flow rate of split injections was 1.0 mL per minute. As a carrier gas, helium at 1.0 mL per minute was used. The MS detector was used in the EI mode with an ionization voltage of 80 eV. The ion source temperature was at 230 °C. The transfer line was at 280 °C. The spectra were collected over the mass range (*m*/*z*) 30–1000. Retention indices were calculated using the retention times of C6-C26 n-alkanes that were injected at the same chromatographic conditions. The volatile constituents were identified by comparison of their relative retention indices and their mass spectra with the Nits 08. L library of essential oil constituents. 

#### 4.5.4. Total Phenols and Antioxidant Activity

Total phenols and antioxidant activity were measured in dry leaf samples for the 2nd cut only during the 2nd season. The air-dried leaves were ground and soaked in methanol. After 24 h, the mixture was filtered, and the filtrate was used to quantify total phenols. The level of total phenols in the crude extracts was determined using the Folin–Ciocalteu reagent and external calibration with gallic acid. Briefly, 0.2 mL of extract solution and 0.2 mL of Folin–Ciocalteu reagent at 755 nm were added, and their contents were mixed thoroughly [99]. After 4 min, 1 mL of 15% Na_2_CO_3_ was added, and then the mixture was allowed to stand for 2 h at normal temperature. The absorbance was measured at 760 nm using a Spectro (Thermo Fisher Scientific, Waltham, MA, USA model 4001/4) spectrophotometer. The concentration of the total phenols was calculated as mg of gallic acid equivalent/g dry weight (D.W) using an equation that was obtained from the gallic acid calibration curve. The determination of total phenolic compounds in the fractions was carried out in triplicate, and the results were averaged [100].

The antioxidative capacity of dry leaves was determined via the 2,2-diphenyl-1-picryl hydrazyl (DPPH) assay [101], with a slight modification. A total of 0.15 mM of 2,2-diphenyl-1-picryl hydra Zyl (DPPH) in 95% ethanol was added to a 0.1% protein solution (in 5 mM poly(1,4-butylene succinate) (PBS) buffer, pH 7.2) in a ratio of 1:1 (*v*/*v*). The mixture was mixed and stored in the dark for 30 min at room temperature. The absorbance of the resulting solution was measured at 517 nm using a spectrophotometer (Helios Gamma; Thermos Fisher Scientific). The blank was prepared in the same manner, except that 5 mM PBS buffer (pH 7.2) was used instead of the sample. The calibration curve was prepared using Trolox in the range from 12.5 to 100 μM. The activity was expressed as nmol Trolox equivalent (TE)/mg dry leaves. 

### 4.6. Statistical Analysis

The data were subjected to an analysis of variance using the SAS program (Version 6.12; SAS Institute Inc., Cary, NC, USA). The mean separations (±SE) were performed using Duncan’s multiple range test through the one-way ANOVA, and significance was determined at *p* ≤ 0.05.

## 5. Conclusions

The findings of our study indicated the advantages of the foliar mode of ZnO NPs, SiO_2_ NPs, and DPPE as partial substitutes for mineral NPK fertilizers. While the ZnO NPs, SiO_2_ NPs, and DPPE at different levels combined with the ¾ or ½ NPK RD demonstrated a significant impact on enhancing the growth traits, essential oil productivity, and biochemical composition of the basil plant relative to the NPK RD in both experimental seasons, the effects of the applied treatments varied across the studied traits. Among these treatments, the most effective treatment was the ½ NPK RD combined with 20 g/L DPPE + 2.0 g/L ZnO NPs in most cases, particularly for the vegetative traits, essential oil %, and yield. Meanwhile, the positive effective treatments on essential oil composition differed and improved such a parameter over the NPK RD, where the identified total compounds ranged from 92.84% to 99.09%, monoterpene hydrocarbons ranged from 0.00 to 1.63%, sesquiterpene hydrocarbons ranged from 11.29 to 25.10%, and oxygenated hydrocarbons ranged from 66.99% to 88.26%. Additionally, the most effective treatments on TPCs and AOA were ¾ NPK + 1.5 g/L ZnO NPs and ½ NPK + 2.0 g/L ZnO NPs, respectively. Such treatments have recorded 12.22 mg GAE/g D.W and 0.03321 µMTE/10 g D.W for TPCs and AOA, respectively, against 8.92 mg GAE/g D.W and 0.02542 µMTE/10 g D.W for the control, respectively. Therefore, the utilization of Zn NPs, Si NPs, and DPPE as alternative sources can potentially reduce the excessive use of traditional NPK fertilizers in order to produce safe medicinal and aromatic plants. Furthermore, the applications of nano essential elements and natural extracts serves important sources to minimize the application of traditional chemical fertilizers, consequently reducing environmental pollution and allowing for the production of safe and healthy products. In addition to the cost of nanoelements, natural extracts are cheap in comparison to mineral fertilizers. Also, the responses of different plant species to nanoelements and natural extracts differ among themselves. Future studies should investigate the effect of different weather conditions and nanotechnology on the production of sweet basil.

## Figures and Tables

**Table 1:** Effect of the fertilization applications on plant height, branch number/plant, main stem diameter (cm), and relative chlorophyll content (RCC) (SPAD units) of sweet basil (average: two cuts) during the 2021 and 2022 seasons.

Fertilization Treatments	Plant Height (cm)		Branch Number/Plants	
	2021	2022	2021	2022
T1—NPK full dose	71.66 ± 1.66 f	72.66 ± 1.32 e	17.33 ± 1.45 b	17.33 ± 0.64 ab
T2—¾ NPK + 10 g/L DPPE	71.33 ± 1.70 f	74.00 ± 1.03 e	13.33 ± 1.62 c	12.66 ± 0.72 d
T3—½ NPK + 20 g/L DPPE	74.66 ± 1.07 e	74.66 ± 1.46 e	20.33 ± 0.76 a	17.00 ± 0.69 ab
T4—¾ NPK + 100 mg/L SiO_2_ NPs	83.00 ± 1.97 b	83.00 ± 1.66 b	15.00 ± 0.94 c	15.00 ± 0.14 c
T5—½ NPK + 150 mg/L SiO_2_ NPs	70.33 ± 0.98 f	73.66 ± 1.51 e	13.33 ± 1.27 c	13.00 ± 0.85 d
T6—¾ NPK + 1.5 g/L ZnO NPs	83.33 ± 1.42 b	83.66 ± 1.20 b	17.66 ± 0.55 b	17.66 ± 0.21 ab
T7—½ NPK + 2.0 g/L ZnO NPs	89.33 ± 1.31 a	92.33 ± 1.65 a	18.33 ± 0.44 b	17.66 ± 0.30 ab
T8—½ NPK + 20 g/L DPPE + 150 mg/L SiO_2_ NPs	79.66 ± 1.34 cd	77.00 ± 1.44 d	21.00 ± 1.25 a	18.00 ± 0.16 a
T9—½ NPK + 20 g/L DPPE + 2 g/L ZnO NPs	78.76 ± 1.82 d	80.33 ± 1.40 c	21.00 ± 0.83 a	19.00 ± 0.89 a
T10—½ NPK + 150 mg/L SiO_2_ NPs + 2 g/L ZnO NPs	81.33 ± 1.73 bc	82.66 ± 1.29 b	18.33 ± 0.33 b	17.33 ± 0.75 ab
	**Main Stem Diameter (cm)**		**Relative Chlorophyll Content (RCC) (SPAD Units)**	
T1—NPK full dose	0.46 ± 0.04 d	0.50 ± 0.04 d	38.66 ± 0.49 e	40.00 ± 0.05 f
T2—¾ NPK + 10 g/L DPPE	0.60 ± 0.08 bc	0.66 ± 0.06 ab	42.66 ± 0.90 c	41.00 ± 0.20 f
T3—½ NPK + 20 g/L DPPE	0.63 ± 0.04 b	0.56 ± 0.15 bcd	43.00 ± 0.91 c	40.66 ± 0.32 f
T4—¾ NPK + 100 mg/LSiO_2_ NPs	0.66 ± 0.03 ab	0.63 ± 0.003 abc	45.00 ± 0.84 b	43.00 ± 0.05 e
T5—½ NPK + 150 mg/L SiO_2_ NPs	0.63 ± 0.03 b	0.63 ± 0.03 abc	43.33 ± 0.87 c	43.00 ± 1.25 e
T6—¾ NPK + 1.5 g/L ZnO NPs	0.50 ± 0.00 d	0.53 ± 0.01 cd	45.00 ± 0.36 b	44.33 ± 0.48 cd
T7—½ NPK + 2.0 g/L ZnO NPs	0.53 ± 0.05 cd	0.56 ± 0.06 bcd	46.00 ± 0.76 ab	47.33 ± 0.58 a
T8—½ NPK + 20 g/LDPPE + 150 mg/L SiO_2_ NPs	0.53 ± 0.05 cd	0.53 ± 0.01 cd	43.66 ± 0.48 c	45.33 ± 1.13 bc
T9—½ NPK + 20 g/L DPPE + 2 g/L ZnO NPs	0.73 ± 0.04 a	0.70 ± 0.02 a	46.66 ± 0.77 a	46.33 ± 0.86 ab
T10—½ NPK + 150 mg/l SiO_2_ NPs + 2 g/LZnO NPs	0.60 ± 0.01 bc	0.60 ± 0.008 abcd	41.33 ± 0.80 d	43.66 ± 0.25 de

Means in each column followed by the same letters are not significantly different at the 0.05 probability level (*p* ≤ 0.05) according to Duncan’s multiple range test (DMRT).

**Table 2:** Effect of the fertilization applications on the fresh and dry weights of aerial parts/plant(g) of sweet basil (average: two cuts) during the 2021 and 2022 seasons.

Fertilization Treatments	Fresh Weights of Aerial Parts/Plant (g)		Dry Weights of Aerial Parts/Plant (g)	
	2021	2022	2021	2022
T1—NPK full dose	821.33 ± 1.52 j	974.00 ± 2.64 i	165.66 ± 1.52 h	165.00 ± 2.64 h
T2—¾ NPK + 10 g/L DPPE	1085.33 ± 1.52 f	980.66 ± 2.12 h	181.33 ± 1.52 f	177.66 ± 2.08 f
T3—½ NPK + 20 g/L DPPE	1274.00 ± 1.73 c	1283.00 ± 2.64 d	210.00 ± 1.73 d	223.33 ± 3.25 c
T4—¾ NPK + 100 mg/L SiO_2_ NPs	965.00 ± 1 h	1242.66 ± 2.51 e	215.66 ± 2.08 c	216.33 ± 3.21 d
T5—½ NPK + 150 mg/L SiO_2_ NPs	949.66 ± 1.52 i	1056.33 ± 2.30 g	180.33 ± 1.52 gf	191.33 ± 2.03 e
T6—¾ NPK + 1.5 g/L ZnO NPs	1059.00 ± 1.11 g	1311.33 ± 2.30 c	190.33 ± 1.52 e	194.66 ± 2.76 e
T7—½ NPK + 2.0 g/L ZnO NPs	1291.66 ± 1.52 b	1442.33 ± 2.51 b	181.33 ± 1.52 f	225.33 ± 2.79 c
T8—½ NPK + 20 g/L DPPE + 150 mg/L SiO_2_ NPs	1202.00 ± 1.73 d	1540.00 ± 2.64 a	239.66 ± 1.52 b	242.66 ± 3.09 b
T9—½ NPK + 20 g/L DPPE + 2 g/L ZnO NPs	1386.66 ± 1.52 a	1541.66 ± 3.05 a	246.33 ± 1.52 a	257.66 ± 2.12 a
T10—½ NPK + 150 mg/L SiO_2_ NPs + 2 g/L ZnO NPs	1165.66 ± 1.15 e	1135.66 ± 2.51 f	178.33 ± 1.74 g	172.66 ± 2.53 g

Means in each column followed by the same letters are not significantly different at the 0.05 probability level (*p* ≤ 0.05) according to Duncan’s multiple range test (DMRT).

**Table 3:** Effect of the fertilization applications on sweet basil EO composition (%) at the 2nd cut during the 2022 season.

	Compound Name (%)	Treatment									
		Control T1	T2	T3	T4	T5	T6	T7	T8	T9	T10
1	Eucalyptol/cineole	5.49	5.04	5.04	0.16	11.47	3.50	5.83	5.44	10.14	5.16
2	Thujone	3.69	1.22	-	0.93	--	-	-	-	-	-
3	Camphor/(+)-2-Bornanone	1.25	0.53	0.48	-	-	0.37	-	-	-	-
4	Estragole	22.25	58.23	46.69	2.12	63.38	23.16	64.5	60.15	46.48	61.19
5	Methyleugenol	47.89	1.20	0.91	0.87	2.85	7.17	1.46	4.29	6.72	5.17
6	Caryophyllene	4.53	7.40	3.39	0.51	4.72	5.69	8.59	8.09	5.45	7.09
7	Cis-a-Bergamotene	1.42	3.60	3.37	0.23	2.10	5.85	2.58	2.70	5.56	2.44
8	Germacrene D	1.53	4.68	2.06	0.75	2.39	3.03	3.37	4.41	2.76	3.05
9	Viridiflorol/ledol	2.40	1.19	-	-	-	-	-	-	-	-
10	(+)-BETA-PINEN	-	0.62	0.51	-	0.43	0.29	0.62	0.40	0.97	0.62
11	1,6-OCTADIEN-3-OL,3,7DIMETHYL-	-	2.54	-	-	-	-	2.05	2.43	-	-
12	Trans-Sesquisabinene hydrate	-	0.51	-	-	-	-	-	-	-	-
13	Ç-Muurolene	-	1.75	0.53	-	0.68	0.68	-	0.68	0.52	-
14	Caryophyllene oxide	-	0.46	2.57	0.44	0.87	3.99	1.15	1.52	0.65	-
15	(-)-Caryophyllene oxide	-	-	-	0.50	-	-	-	-	-	2.79
16	p-Cymene	-	-	0.51	-	-	0.44	-	-	-	-
17	Terpineol	-	-	0.65	-	-	0.72	-	-	-	-
18	Terpinyl	-	-	0.48	-	-	-	-	-	-	-
19	Terpinen	-	-	-	-	-	-	-	-	1.12	0.49
20	Linalool	-	-	19.68	-	3.31	20.44	-	-	10.64	2.94
21	Linalool oxide	-	-	-	-	-	0.43	-	-	-	-
22	Cis-Geraniol	-	-	1.07	-	-	1.05	-	-	-	-
23	Cis-Verbenol	-	-	1.52	-	-	-	-	-	-	-
24	Cis-à-Bisabolene	-	-	2.55	0.72	-	0.41	-	-	-	-
25	Citral/à-Citral	-	-	1.34	-	-	2.32	-	-	-	-
26	Epiglobulol	-	-	-	0.82	-	0.55	-	-	-	-
27	Á-Pinene	-	-	-	-	1.20	-	-	-	-	-
28	Ocimene	-	-	-	-	-	-	-	-	0.34	
29	Cis-ocimene	-	-	-	-	-	0.28	-	-	-	
30	A-Humulene/a-Caryophyllene	-	-	1.36	0.09	-	2.60	3.86	3.89	2.50	3.42
31	1H-Benzocycloheptene, 2,4a,5,6,7,8,9,9a-octahydro-3,5,5-trimethyl-9-methylene-, (4aS-cis)-	3.03	6.30	-	-	-	-	-	-	-	-
32	1-Naphthalenepropanol, à-ethenyldecahydro-à,5,5,8a-tetrame thyl-2-methylene-, [1S-[1à(R*),4aá,8aà]]-	2.58	1.03	-	0.85	--	-	-	-	-	-
33	3-CYCLOHEXEN-1-OL, 4-METHYL-1-(1-METHYLETHYL	-	-	3.07	-	-	3.00	-	-	-	-
34	CYCLOHEXENE,4-(1,5 DIMETHYL-1,4-HEXADIENYL)-1-METHYL-	-	-	-	-	3.69	4.41	5.54	5.55	3.85	5.05
35	Cadina	-	-	-	-	-	0.33	-	-	-	-
36	Epicubenol	-	-	-	-	-	0.33	-	-	-	-
37	Patchoulene	-	-	-	-	-	0.37	-	-	-	-
38	BETA-ELEMEN	-	-	-	-	-	0.51	-	-	-	-
39	(−)-á-Bourbonene	-	-	-	-	-	0.21	-	-	-	-
40	Nerol acetate	-	-	-	-	-	0.39	-	-	-	-
41	Copaene	-	-	-	-	-	0.32	-	-	-	-
42	À-acorenol	-	-	-	-	-	-	0.44	0.43	0.43	-
43	Doconexent	-	-	-	-	-	-	-	-	0.29	-
44	HUMULADIENONE	-	-	-	-	-	-	-	-	-	0.58
Total compounds (%)		96.06	96.3	97.78	99.99	97.09	92.84	99.99	99.98	98.42	99.99
Monoterpene hydrocarbons (%)		0.00	0.62	0.9	0.44	1.63	1.01	0.62	0.4	1.31	0.62
Sesquiterpenes hydrocarbons (%)		13.28	20.07	13.26	11.29	12.09	24.84	24.39	25.10	21.16	21.05
Oxygenated hydrocarbons (%)		82.78	75.61	83.62	88.26	83.37	66.99	74.98	74.48	75.95	78.32

T1—NPK full dose, T2—¾ NPK + 10 g/L DPPE, T3—½ NPK + 20 g/L DPPE, T4—¾ NPK + 100 mg/L SiO_2_ NPs, T5—½ NPK + 150 mg/L SiO_2_ NPs, T6—¾ NPK + 1.5 g/L ZnO NPs, T7—½ NPK + 2.0 g/L ZnO NPs, T8—½ NPK + 20 g/L DPPE + 150 mg/L SiO_2_ NPs, T9—½ NPK + 20 g/L DPPE + 2 g/L ZnO NPs, and T10—½ NPK + 150 mg/L SiO_2_ NPs + 2 g/L ZnO NPs.

**Table 4:** Effect of the fertilization applications on sweet basil total phenolic compounds and antioxidant activity at the 2nd cut during the 2022 season.

Fertilization Treatments	Total Phenols (mg GAE/g D.W)	Antioxidant Activity (µM TE/10 g D.W)
	2021	2021
T1—NPK full dose	8.92 ± 0.01 h	0.02542 ± 0.00 f
T2—¾ NPK + 10 g/L DPPE	6.52 ± 0.01 j	0.02804 ± 0.00 e
T3—½ NPK + 20 g/L DPPE	8.32 ± 0.01 i	0.02864 ± 0.00 ed
T4—¾ NPK + 100 mg/L SiO_2_ NPs	9.61 ± 0.00 f	0.02813 ± 0.00 e
T5—½ NPK + 150 mg/L SiO_2_ NPs	10.10 ± 0.01 e	0.03033 ± 0.00 b
T6—¾ NPK + 1.5 g/L ZnO NPs	12.22 ± 0.00 a	0.02940 ± 0.00 bcd
T7—½ NPK + 2.0 g/L ZnO NPs	11.25 ± 0.01 b	0.03321 ± 0.00 a
T8—½ NPK + 20 g/L DPPE + 150 mg/L SiO_2_ NPs	10.83 ± 0.00 d	0.02991 ± 0.00 cb
T9—½ NPK + 20 g/L DPPE + 2 g/L ZnO NPs	9.08 ± 0.01 g	0.02889 ± 0.00 cde
T10—½ NPK + 150 mg/L SiO_2_ NPs + 2 g/L ZnO NPs	11.20 ± 0.01 c	0.02948 ± 0.00 bcd

Means in a column that have the same letters are not significantly different (*p* ≤ 0.05) according to DMRT.

**Table 5:** The specification, physico-chemical properties, and acute toxicity of ZnO NPs and SiO_2_ NPs.

	Silica Oxide Nanoparticles	Zinc Oxide Nanoparticles
	Specification	
Appearance	White powder	White powder
Average particle size	15 ± 10 nm	20 nm
Morphology	Spherical	Spherical
Surface area	109.356 m^2^/g	2.7534 m^2^/g
Average pore radius	3.53198 × 10^1^ Å	40.5965 nm
Total pore volume	1.931 × 10^−2^ cc/g	0.042062 cm^3^/g
Chemical composition	silicon = 46.83%; oxygen = 53.33%	Zn = 80.34%; O = 19.6
Acute toxicity		
	Inhalation human LD50 = 3000 mg/kg Intravenous rat LD50 = 90 mg/kg Intravenous mouse LD50 = 40 mg/kg Oral rat LD50 > 3000 mg/kg Dermal rabbit LD50 > 5000 mg/kg	The lethal dose 50 (LD50) of intravenously administration = 0.3 mg/kg in mice The LD50 of intratracheal instillation = 493.85 µg/kg in mice

## Data Availability

All data generated or analyzed during this study are included in this published article.
